# Supplementary material for: Metabolomics of in vivo inflammation identifies soluble sialic acid as a conserved myeloid-cell metabolite
Source: bioRxiv. 2026 Jul 7:2026.07.06.736822. Preprint. [Version 1] doi: 10.64898/2026.07.06.736822 (PMC13370421; doi:10.64898/2026.07.06.736822)
Supplement: Supplement 1 [file NIHPP2026.07.06.736822v1-supplement-1.pdf]

bioRxiv preprint doi: https://doi.org/10.64898/2026.07.06.736822; this version posted July 7, 2026. The copyright holder for this preprint (which was not certified by peer review) is the author/funder, who has granted bioRxiv a license to display the preprint in perpetuity. It is made available under a CC-BY 4.0 International license.

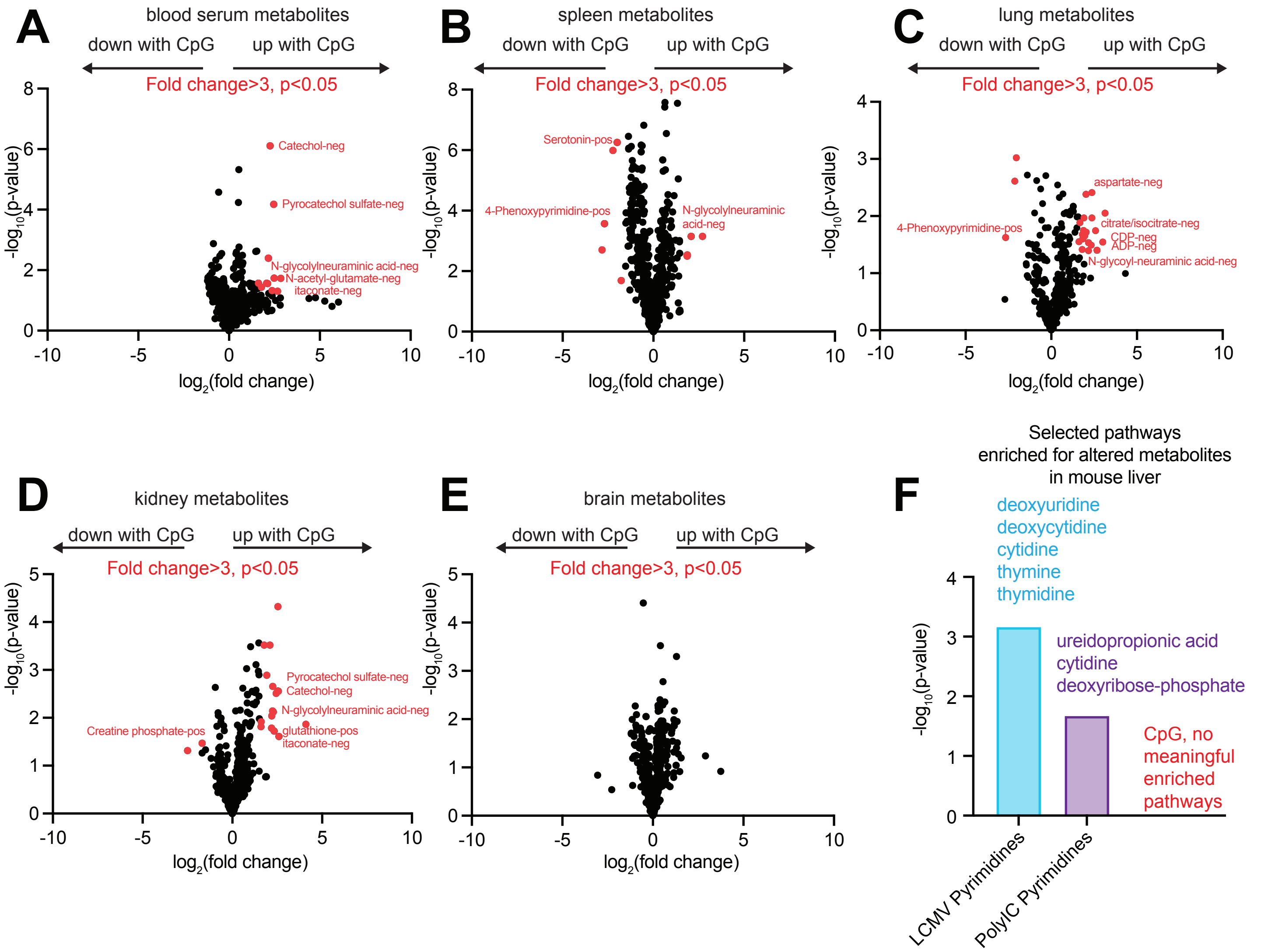

Figure S1, related to Figure 1: Metabolomic changes and pathway enrichments in inflamed mouse tissues. A-E: Metabolites changed between control mice and mice receiving intraperitoneal injection of 50 µg CpG DNA oligonucleotide (or control oligonucleotide) on days 0,2,4,6,8, with tissue collection on day 10, n=4 mice per group. (A) metabolites in blood serum, (B) spleen, (C) lung, (D) kidney, (E) brain; metabolites meeting the threshold of greater than 3-fold or less than 0.33-fold change, and $p<0.05$, are highlighted in red and selected metabolites are labeled, p-values were determined using a two-sided t test. (F) Pathways enriched for altered metabolites in mouse liver after 8 days of LCMV Clone 13 infection, 24 hours after polyI:C injection, or after 10 days of CpG injection; data corresponds to Figure 1. All enriched pathways with more than 2 metabolites represented are shown.

bioRxiv preprint doi: https://doi.org/10.64898/2026.07.06.736822; this version posted July 7, 2026. The copyright holder for this preprint (which was not certified by peer review) is the author/funder, who has granted bioRxiv a license to display the preprint in perpetuity. It is made available under a CC-BY 4.0 International license.

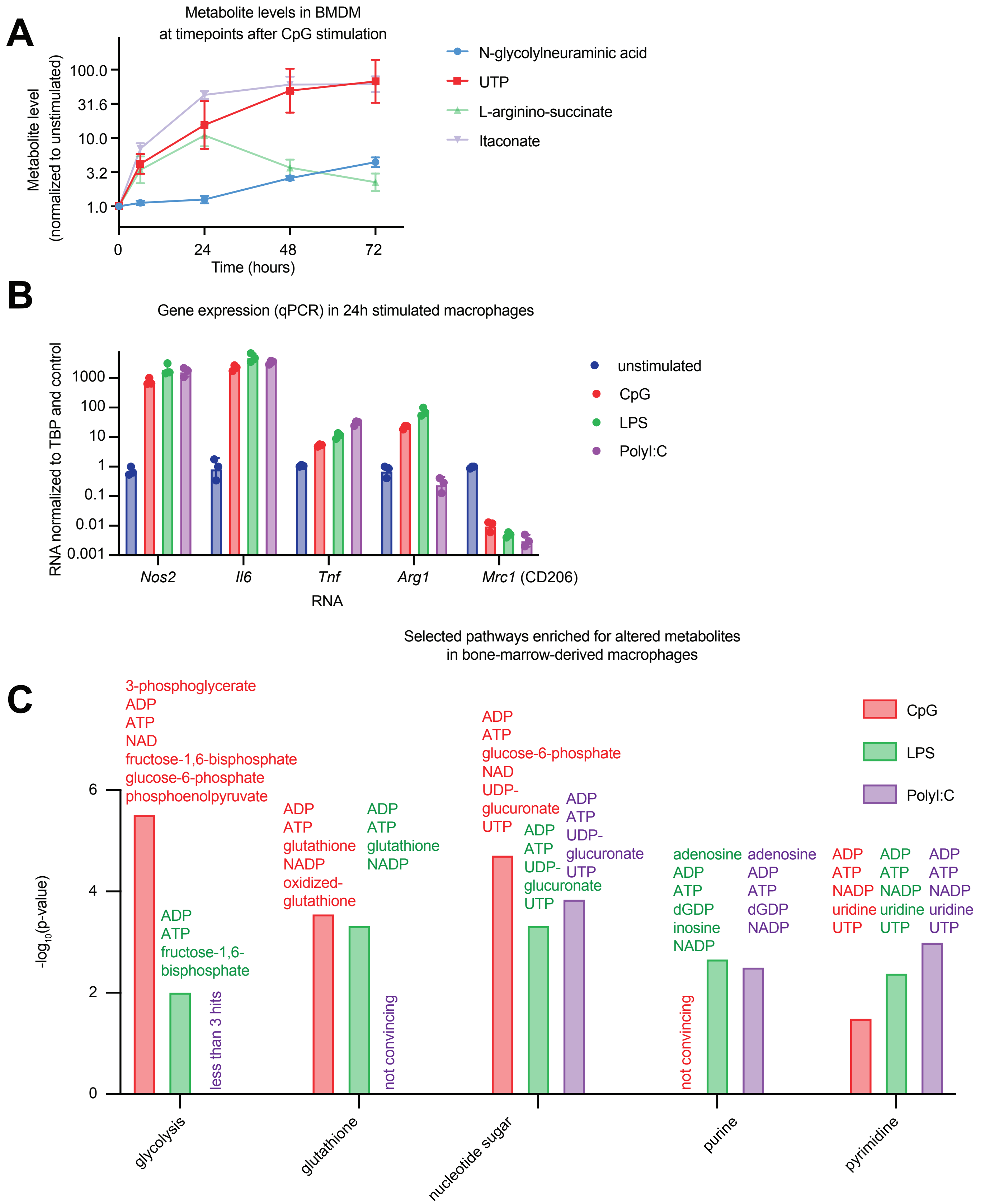

Figure S2, related to Figure 2: Metabolomic changes, gene expression changes, and pathway enrichments in stimulated bone-marrow-derived macrophages.A: Changes in selected metabolites at 0,4,24,48 or 72 hours after stimulation with 4ng/mL CpG oligonucleotide, showing that 24 hours is an early timepoint where large metabolite changes are detected. B: Quantitative PCR for selected genes in bone-marrow-derived macrophages 24 hours after stimulation with 4 ng/mL CpG DNA oligonucleotide, 4 ng/mL lipopolysaccharide (LPS), or 500ng/mL polyinosinic-polycytidylic acid (PolyI:C). C: Pathways enriched for altered metabolites in macrophages after 24 hours of stimulation with CpG, LPS or PolyI:C, data corresponds to Figure 2. Selected enriched pathways with more than 2 metabolites represented.

bioRxiv preprint doi: https://doi.org/10.64898/2026.07.06.736822; this version posted July 7, 2026. The copyright holder for this preprint (which was not certified by peer review) is the author/funder, who has granted bioRxiv a license to display the preprint in perpetuity. It is made available under a CC-BY 4.0 International license.

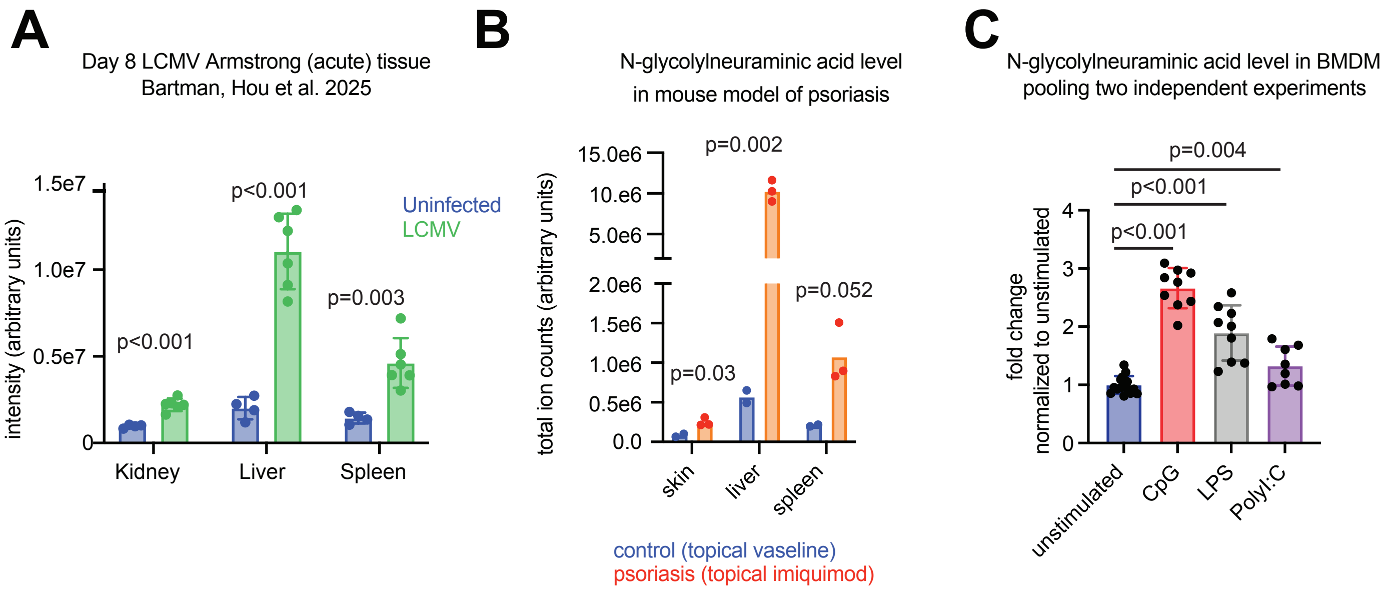

Figure S3, related to Figure 3: Inflammation increases N-glycolylneuraminic acid more in mouse tissues than in cultured macrophages.A: N-glycolylneuraminic acid level from kidney, liver and lung of mice infected with acute Armstrong LCMV for 8 days, n=4 uninfected and n=6 infected mice, data from Bartman, Hou et al. 202545. B: N-glycolylneuraminic acid level in mouse skin, liver and spleen induced by 7 days topical application of 60mg imiquimod or vaseline control, n=2 control and n=3 imiquimod-treated mice. C: N-glycolylneuraminic acid level in bone-marrow-derived macrophages 24 hours after stimulation with 4 ng/mL CpG DNA oligonucleotide, 4 ng/mL lipopolysaccharide (LPS), or 500ng/mL polyinosinic-polycytidylic acid (PolyI:C), pooling two independent experiments, each normalized to unstimulated macrophages from same experiment.
All p-values from two-sided t-tests.

bioRxiv preprint doi: https://doi.org/10.64898/2026.07.06.736822; this version posted July 7, 2026. The copyright holder for this preprint (which was not certified by peer review) is the author/funder, who has granted bioRxiv a license to display the preprint in perpetuity. It is made available under a CC-BY 4.0 International license.

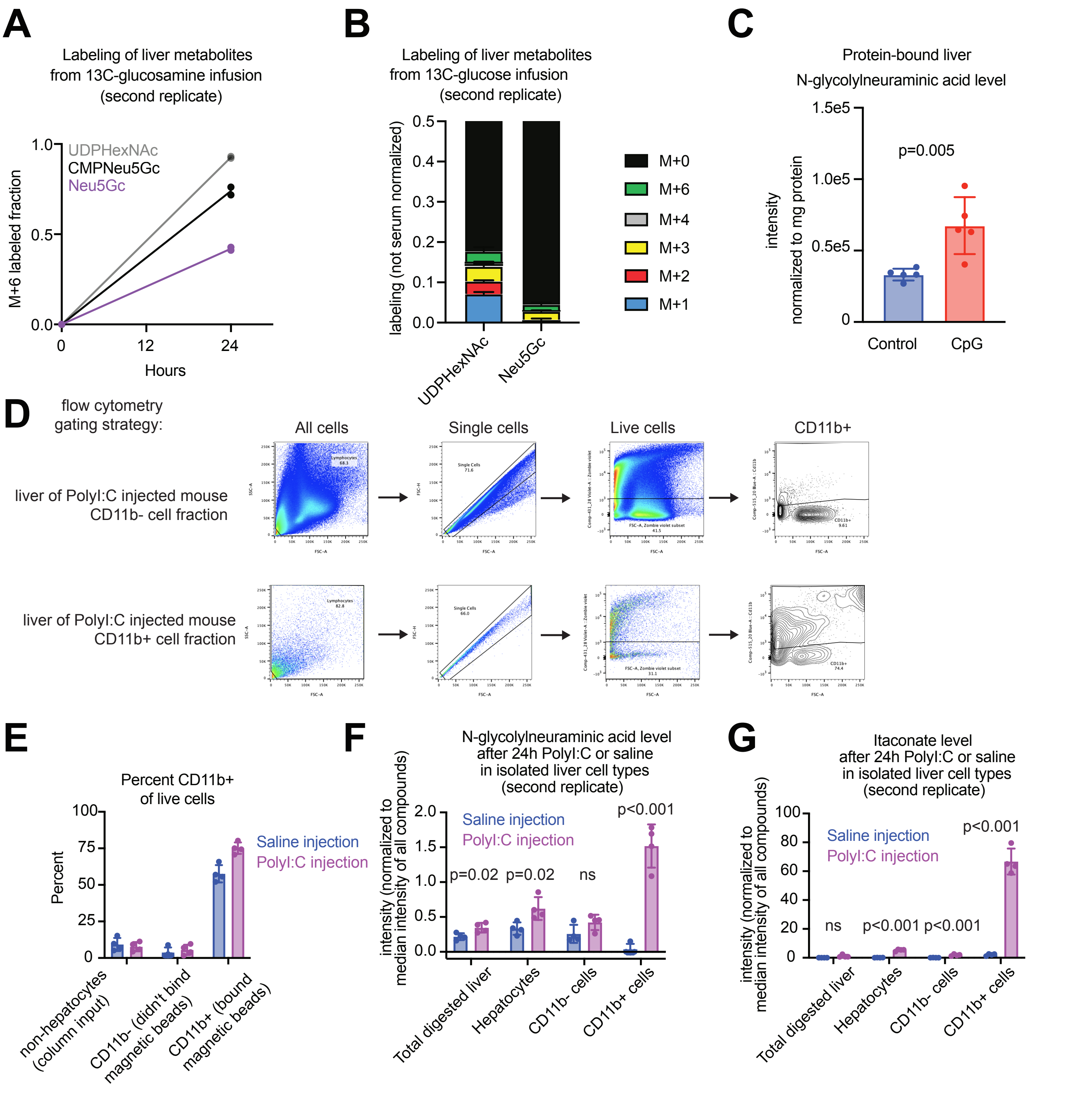

Figure S4, related to Figure 4: N-glycolylneuraminic acid is produced from sialylated proteins by CD11b+ myeloid immune cells in vivo. A : Mass+6 (m+6) fractional carbon-13 labeling of UDP-HexNAc, CMP-Neu5Gc, and free soluble Neu5Gc in mouse liver after continuous infusion of m+6 13C-glucosamine (m+6) 0 or 24 hours, n=1 uninfused and n=2 24h-infused mice. B: Carbon-13 isotopologue distribution (m+0 through m+6) for UDP-HexNAc and soluble Neu5Gc after 24 hours of m+6 13C-glucose infusion, n=2 mice. C: Protein-bound Neu5Gc measured by protein isolation from mouse livers and hydrolysis with acetic acid, in mice injected with control oligonucleotide or with CpG over 10 days, or with both CpG and 30mg/kg P-3FAX-Neu5Ac sialyltransferase inhibitor, n=5 mice per group. D-E: Flow cytometry data to assess purity of liver CD11b+ myeloid cell isolation, related to data in Figure 4I. F: N-glycolylneuraminic acid level normalized to the median intensity of all metabolites in whole liver, isolated hepatocytes, CD11b− non-hepatocytes, and CD11b+ cells from control and PolyI:C-treated mice, n = 4 mice per group, second independent replicate of experiment in Figure 4I. G: Itaconate level normalized to the median intensity of all metabolites in whole liver, isolated hepatocytes, CD11b− non-hepatocytes, and CD11b+ cells from control and PolyI:C-treated mice, n = 4 mice per group. As expected, itaconate is enriched in CD11b+ myeloid cells. All p-values from two-sided t-tests.

bioRxiv preprint doi: https://doi.org/10.64898/2026.07.06.736822; this version posted July 7, 2026. The copyright holder for this preprint (which was not certified by peer review) is the author/funder, who has granted bioRxiv a license to display the preprint in perpetuity. It is made available under a CC-BY 4.0 International license.

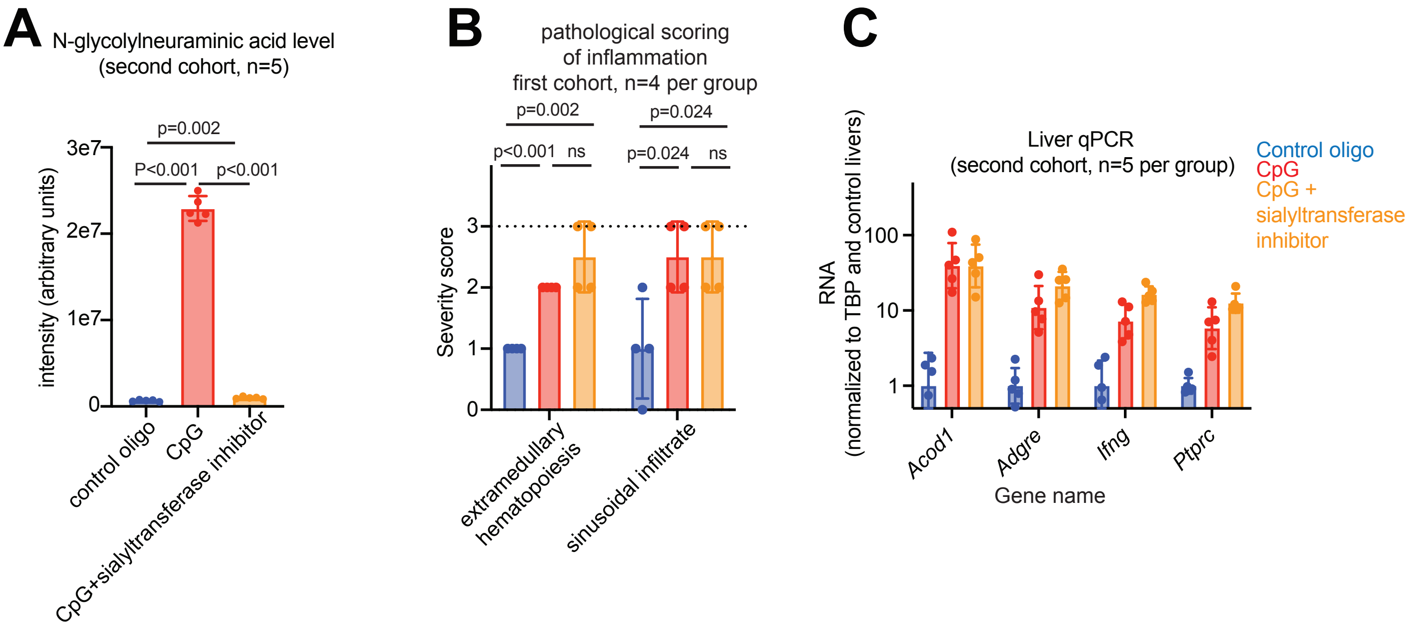

Figure S5, related to Figure 5: N-glycolylneuraminic acid does not affect pathology in CpG-induced cytokine storm. A : Soluble N-glycolylneuraminic acid level in livers of mice injected over 10 days with control oligonucleotide, CpG oligonucleotide, or CpG plus daily 30mg/kg P-3FAX-Neu5Ac sialyltransferase inhibitor injection, n=5 mice per group. B: Blinded pathologist scoring of liver inflammation in mice injected over 10 days with control oligonucleotide, CpG oligonucleotide, or CpG plus 30mg/kg P-3FAX-Neu5Ac sialyltransferase inhibitor, n=4 mice per group, second independent replicate of experiment in Figure 5C. C: Quantitative PCR of selected RNAs in livers of mice from Figure S5A, n=5 mice per group. in All p-values from two-sided t-tests.

bioRxiv preprint doi: https://doi.org/10.64898/2026.07.06.736822; this version posted July 7, 2026. The copyright holder for this preprint (which was not certified by peer review) is the author/funder, who has granted bioRxiv a license to display the preprint in perpetuity. It is made available under a CC-BY 4.0 International license.

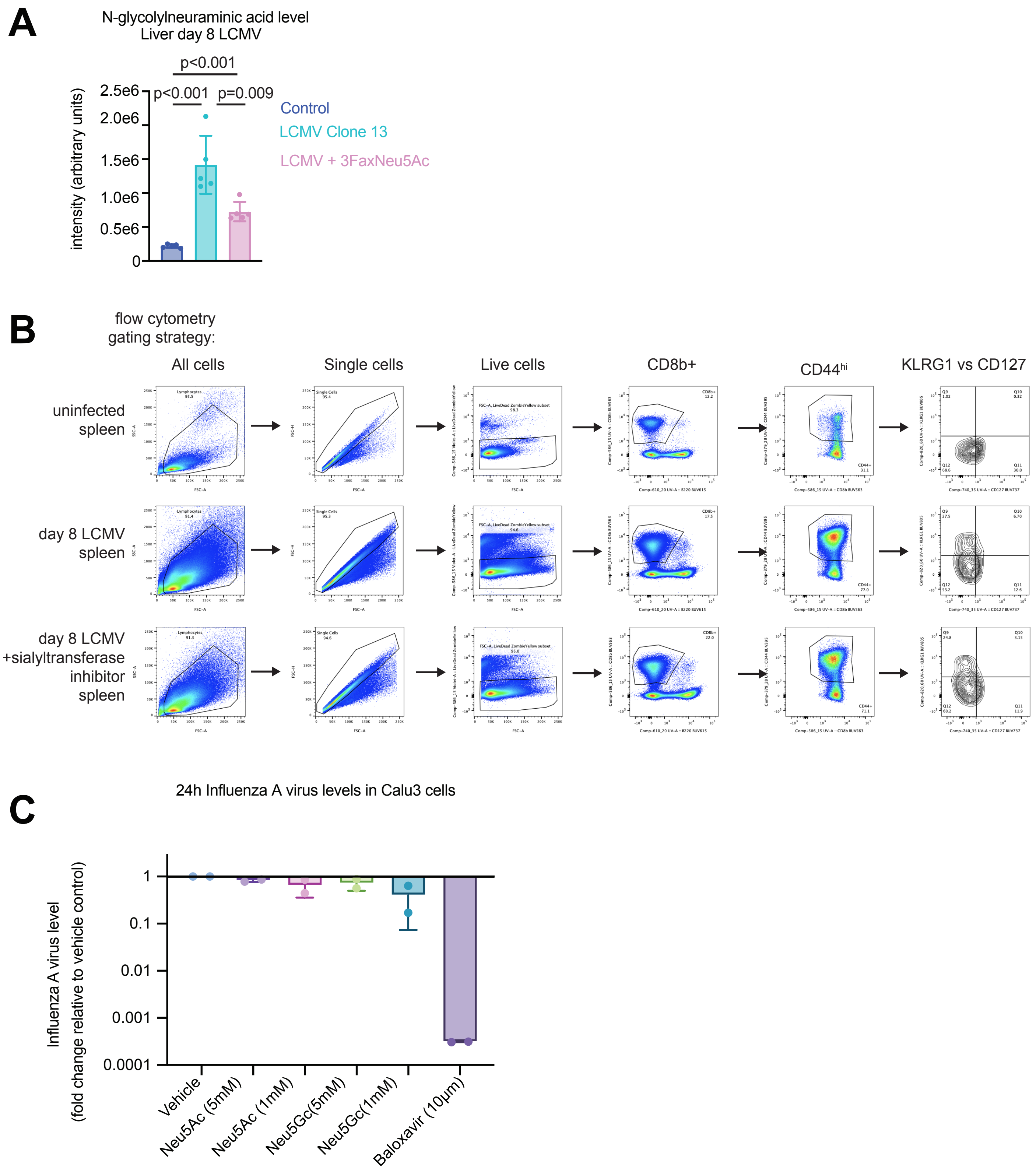

Figure S6, related to Figure 5: N-glycolylneuraminic acid does not affect viral infection.
A: Soluble N-glycolylneuraminic acid level in livers of uninfected mice, mice 8 days after LCMV Clone 13 infection, or LCMV Clone 13 plus daily 30mg/kg P-3FAX-Neu5Ac sialyltransferase inhibitor injection, n=5 mice per group, p-values from two-sided t-tests. B: Flow cytometry gating strategy to analyze CD8+ T cell phenotype, related to Figure 5G-H. C. Soluble N-glycolylneuraminic acid and N-acetylneuraminic acid do not reduce Influenza A virus infection in Calu-3 lung epithelial cells, showing n=2 independent experiments, each an average of 3 wells. Baloxavir (Influenza virus endonuclease inhibitor) is a positive control.
